# An Improved RNA Extraction Protocol for Rye Grain Full-Length Transcriptome Sequencing

**DOI:** 10.3390/ijms252313188

**Published:** 2024-12-08

**Authors:** Justyna Jazowska, Mateusz Przyborowski, Marek Wojciechowski, Jolanta Groszyk

**Affiliations:** Plant Breeding and Acclimatization Institute–National Research Institute, Radzików, 05-870 Błonie, Poland; j.jazowska@ihar.edu.pl (J.J.); m.przyborowski@ihar.edu.pl (M.P.); m.wojciechowski@ihar.edu.pl (M.W.)

**Keywords:** ONTs, LiSTE, IHSP, grain, starch, RNA, RIN, rye, *Secale cereale*

## Abstract

RNA quality and integrity are critical for many studies in plant molecular biology. However, extracting high-quality RNA from cereal grains is challenging due to the presence of polysaccharides, polyphenols, and other compounds that bind or coprecipitate with RNA particles. To address this, we introduced an initial purification step into the Tri Reagent Solution protocol, which effectively eliminated starch and other contaminants. The performance of this modified protocol was then compared with five other RNA extraction methods, including those based on Tri Reagent Solution and the Direct-zol RNA Miniprep Plus Kit. Introducing our method modification prior to homogenization with Tri Reagent Solution successfully yielded total RNA with both high quality (RIN values ranging from 9.50 to 9.70) and high efficiency, making it suitable for both mRNA extraction using the Dynabeads mRNA Purification Kit and library preparation for transcriptome sequencing by long-read methods, such as Oxford Nanopore Technologies. The protocol was successfully applied to total RNA extraction from rye grains at 14 and 21 days after pollination. This study demonstrates that improving the Tri Reagent Solution protocol through initial purification enables the extraction of high-quality RNA from rye grains.

## 1. Introduction

Nowadays, transcriptome analysis relies heavily on methods utilizing both second- (Illumina) and third-generation (Pacific Biosciences (PacBio), Oxford Nanopore Technologies (ONTs)) sequencing technologies. The manufacturer ONTs yields reads of any length that can span full-length RNA transcripts. Therefore, obtaining high-quality, undegraded total RNA that is devoid of contaminants is critical for successful mRNA extraction and subsequent library preparation to yield representative, full-length molecules [1]. This is commonly assessed spectrophotometrically, and the recommended sample quality parameters evaluated include the A_260_/A_280_ ratio (1.8–2.0), the A_260_/A_230_ ratio (2.0–2.2), and the total RNA concentration. A_260_/A_280_ ratio lower than ~2.0 may indicate DNA contamination, while a ratio lower than ~1.8 suggests the presence of proteins. A significantly lower A_260_/A_230_ ratio (<2.0) indicates the presence of contaminants such as carbohydrates or residual phenol. These contaminants can act as PCR inhibitors and can reduce the efficiency of library preparation steps; hence, total RNA samples may require additional purification steps to ensure efficient library synthesis and avoid subsequent degradation. Total RNA quality can also be assessed using automated electrophoresis systems like the Agilent 2100 Bioanalyzer or Agilent TapeStation. These systems provide RNA Integrity Number (RIN) or RNA Integrity Number equivalent (RINe) values, respectively. High-quality, full-length total RNA typically has an RIN score of 9.5, while degraded samples often have RIN values below 4.5, highlighting the need to optimize experimental setups for specific tissues and final sample concentrations used in subsequent analyses. Interestingly, low RIN values observed in total RNA extracted from mature embryos of long-term stored barley (*Hordeum vulgare* L.) samples did not necessarily indicate poor mRNA quality [2].

**Figure 1:**
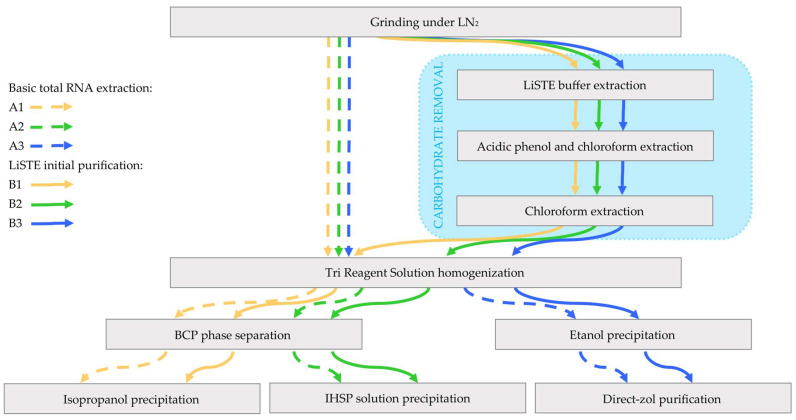
Experiment flowchart illustrating the total RNA extraction with Tri Reagent Solution (Invitrogen, Waltham, MA, USA) and Direct-zol RNA Miniprep Plus Kit (Zymo Research Corporation, Irvine, CA, USA). LN_2_—liquid nitrogen; LiSTE—extraction buffer contains 150 mM LiCl, 1% SDS, 50 mM Tris-HCl (pH 8.0), and 5 mM EDTA (pH 8.0); BCP—1-bromo-3-chloropropane; IHSP—isopropanol and high-salt precipitation contains isopropanol and 1.2 mol/L NaCl with 0.8 mol/L sodium citrate in a 1:1 ratio.

During their maturation, cereal grains accumulate starch and other polysaccharides [3,4], which can significantly reduce the quality of the extracted RNA. While many of the commercial reagents and kits that are available can achieve the desired quality and quantity of total RNA extracted, they often require lengthy trial and error optimization steps for each species type and seed age. Here, we present an optimized, SDS-based protocol for total RNA extraction combined with a commercial TRI Reagent Solution (Invitrogen, Waltham, MA, USA) and compare it with the standard manufacturer’s protocol, as well as with the Direct-zol RNA Miniprep Plus Kit (Zymo Research Corporation, Irvine, CA, USA) (Figure 1). SDS extraction-based methods described by Prescott and Martin [5] have been successfully used for total RNA extraction from tobacco (*Nicotiana tabacum* L.) leaves [6], Antirrhinum (*Antirrhinum majus* L.) flowers [7], carrots (*Daucus carota* L.) [8], and Arabidopsis (*Arabidopsis thaliana* L.) [9,10]. Modified versions of these methods have been applied for total RNA extraction from triticale (×*Triticosecale* Wittm.), wheat (*Triticum aestivum* L.) [11], and barley [12].

Aiming to achieve high-quality total RNA for transcriptome sequencing by ONTs, we tested both the standard and modified protocols for total RNA extraction from grains based on Tri Reagent Solution and the Direct-zol RNA Miniprep Plus Kit. Due to the high concentration of polysaccharides, polyphenols, and other compounds that bind or coprecipitate with the RNA in rye (*Secale cereale* L.) grains, we enriched the Tri Reagent Solution-based protocol with an initial purification step of plant tissue before homogenization. This approach was compared to five other total RNA extraction protocols using Tri Reagent Solution (Figure 1), which have been previously reported as successful for total RNA extraction from cereals.

## 2. Results and Discussion

### 2.1. Protocols for High-Quality Total RNA Extraction

**Figure 2:**
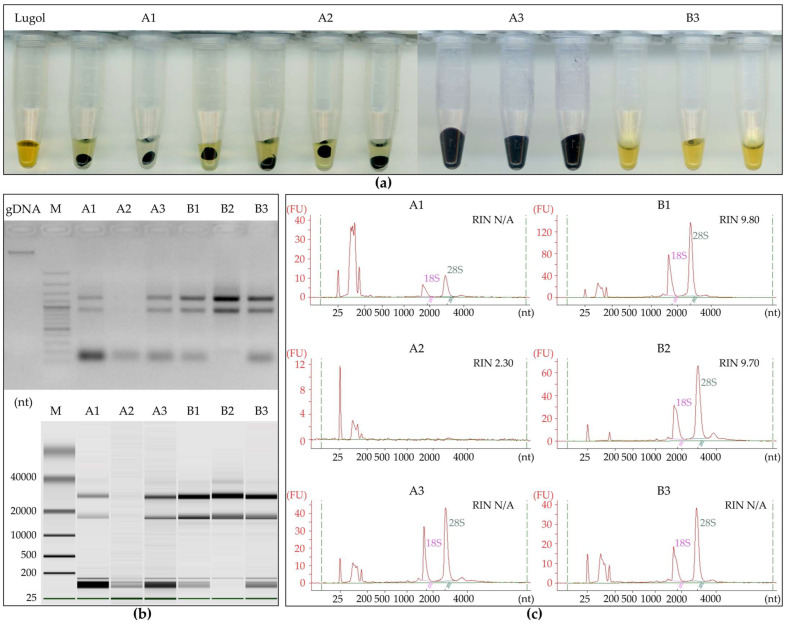
Results of quality and quantity control of total RNA extracted using six protocols. (**a**) Visualization of starch in total RNA preps using 7% Lugol solutions (tube 1); pellets from protocols A1 (tubes 2–4) and A2 (tubes 5–7); and suspensions from protocols A3 (tubes 8–10) and B3 (tubes 11–13); (**b**) electrophoresis 500 ng of genomic DNA (gDNA) and 1 µg total RNA run on a 1% TAE-agarose gel with GeneRuler 100 bp Plus DNA Ladder (Thermo Fisher Scientific Baltics UAB, Vilnius, Lithuania) (upper panel) and Bioanalyzer 2100 RNA 6000 Nano Kit gel-like images using 200–450 ng total RNA (lower panel); (**c**) Agilent 2100 Bioanalyzer chromatograms of total RNA, showing markers (25 nt), small RNA fraction (≥100 nt), 5S rRNA, and degraded 18S rRNA and 28 S rRNA subunits (100–150 nt), 18 S rRNA subunit (≥2000), the 28S rRNA subunit (≈3000), and RNA Integrity Number (RIN). Detailed parameters for all samples are shown in Table S1.

In our experiments, all the tested protocols successfully extracted total RNA but differed in terms of both yield and quantity (Table 1). The standard total RNA extraction protocol, which includes sample grinding in liquid nitrogen, homogenization with Tri Reagent Solution (Invitrogen, Waltham, MA, USA), phase separation using 1-bromo-3-chloropropane (BCP) or chloroform, precipitation with isopropanol, and washing and dissolving total RNA in water, has proven efficient for young tissues of cereal species such as barley (*Hordeum vulgare* L.) leaves and roots [13,14] and mature embryos [2]. However, when applied to rye (*Secale cereale* L.) grains, this protocol resulted in undissolved white precipitates both in standard isopropanol precipitation (A1) as well as in isopropanol and a high-salt precipitation (IHSP) solution containing 1.2 mol/L NaCl and 0.8 mol/L sodium citrate recommended by the manufacturer for polysaccharide-rich tissue (A2). These pellets were stained dark blue Lugol’s solution, indicating the presence of significant amounts of starch (Figure 2a). On the other hand, the supernatant showed total RNA concentrations of 148–190 ng/µL, with an A_260_/A_280_ ratio within the acceptable range, but an excessively low A_260_/A_230_ ratio in samples precipitated with isopropanol (Table 1). Precipitation using IHSP solution resulted in lower total RNA concentrations and poorer absorbance ratios than isopropanol alone (Table 1).

Column-based methods are effective for total RNA extraction from cereal species across developmental stages [15,16,17]. The Direct-zol RNA Miniprep Plus Kit (Zymo Research Corporation, Irvine, CA, USA) was applied for total RNA extraction from wheat (*Triticum aestivum* L.) leaves [18], and for maize (*Zea mays* L.), seed soak water was applied to detect maize chlorotic mottle virus (MCMV) [19]. These methods generally reduce processing time, are technically simpler in some cases, and result in high A_260_/A_280_ and A_260_/A_230_ ratios, though with lower final total RNA yields. Despite additional centrifugation and starch precipitation before column purification (Figure 2a), utilizing the Direct-zol RNA Miniprep Plus Kit for rye grain total RNA extraction (A3) resulted in eluted RNA of both low quantity and quality (Table 1).

Aiming to improve total RNA yield and purity, we modified the protocol by introducing an extraction step with LiSTE buffer containing 150 mM LiCl, 1% Sodium Dodecyl Sulfate (SDS), 50 mM Tris-HCl (pH 8.0), and 5 mM EDTA (pH 8.0), and for the initial purification of plant tissue prior to homogenization with Tri Reagent Solution (B1, B2, B3). All purification and centrifugation steps were carried out either in an ice bath or at 4 °C. Ground grain incubation in LiSTE buffer, followed by an extraction with acidic phenol and chloroform, resulted in the removal of most carbohydrates and cell debris. The low pH (4.3) of the buffer also helped eliminate high amounts of DNA (which migrated to the alkaline organic phase). Re-extraction of the aqueous phase with chloroform further purified the solution used for homogenization with Tri Reagent Solution. In the precipitation step, isopropanol effectively precipitated total RNA, which was observed as a clear pellet after centrifugation, and finally, it fully dissolved in water (B1). This protocol provided the best results among all the tested protocols (Table 1). The RNA quantity was over 2.6-fold higher than A1, and its quality and integrity, measured spectrophotometrically (DeNovix DS-11) and native electrophoresis in 1% TAE-agarose gel, and confirmed by automated electrophoresis (Agilent 2100 Bioanalyzer), were excellent (Figure 2b). Precipitation using IHSP solution (B2) produced appropriate A_260_/A_280_ ratios but low A_260_/A_230_ ratios (Table 1). This method, which included ethanol precipitation and centrifugation before column loading (B3), resulted in minimal starch contamination (Figure 2a) and yielded high-quality total RNA (Table 1). In both protocols (B1 and B3), the extracted total RNA was free of protein and secondary compounds, as indicated by the A_260_/A_280_ and A_260_/A_230_ ratios. RNA integrity analysis performed by the total RNA assay with Agilent 2100 Bioanalyzer and Bioanalyzer 2100 RNA 6000 Nano Kit showed the high integrity of total RNA extracted using the B1 and B2 protocol, which had a 9.80 and 9.70 RIN value, respectively (Figure 2c). In both protocols, 18S and 28S rRNA had high concentrations, while 5S rRNA was detected in the B1 protocol. Similarly, 5S rRNA was detected in the B3 protocol. Using an IHSP solution for total RNA precipitation eliminated low-molecular-weight RNA molecules (e.g., 5S rRNA, miRNA, tRNA) (Figure 2c).

The B1 protocol based on the homogenization of the sample tissue with LiSTE buffer at 4 °C (centrifugation) and an ice bath (incubation) causes a decrease in RNase activity. Despite the increase in the number of steps in the B1 protocol compared to the A1 and A2 protocols, the cost of reagents and materials is only slightly higher. In the case of protocols A3 and B3, the Direct-zol RNA Miniprep Plus Kit columns incur a more significant cost. An analogous situation is the case of the time required for RNA extraction. Protocols with initial purification steps (B1, B2, and B3) are prolonged with additional steps, about 30–40 min. On the other hand, in our opinion, the use of columns significantly speeds up the extraction of RNA from TRI Reagent Solution due to the reduction in centrifugation time from 10 min to an average of 1 min.

### 2.2. Inhibition Test

**Figure 3:**
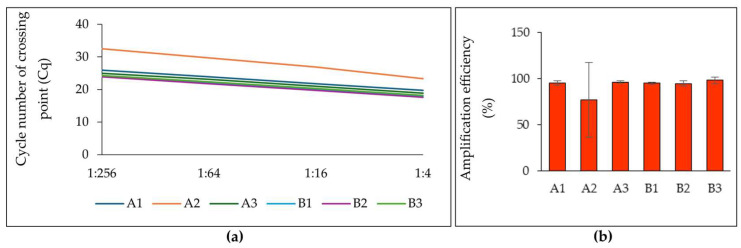
Results of PCR inhibitors test performed using total RNA. (**a**) Cycle number of crossing point for dilutions determined in One-Step RT-qPCR; (**b**) Amplification efficiency determined by One-Step RT-qPCR for different protocol of total RNA extractions.

To assess RNA quality, replicates of extracted total RNA were tested for RT-qPCR inhibition according to the published criteria [20]. Briefly, sample serial dilutions were used for amplifying the reference genes using the Luna Universal One-Step RT-qPCR Kit (New England Biolabs, Ipswich, MA, USA). These results were used to calculate the amplification efficiency (Figure 3a,b). Among the tested extraction methods, protocol B1 was the most consistent between the extraction replicates, while also achieving optimal amplification efficiency.

### 2.3. Transcriptome Sequencing by Oxford Nanopore Technologies

**Figure 4:**
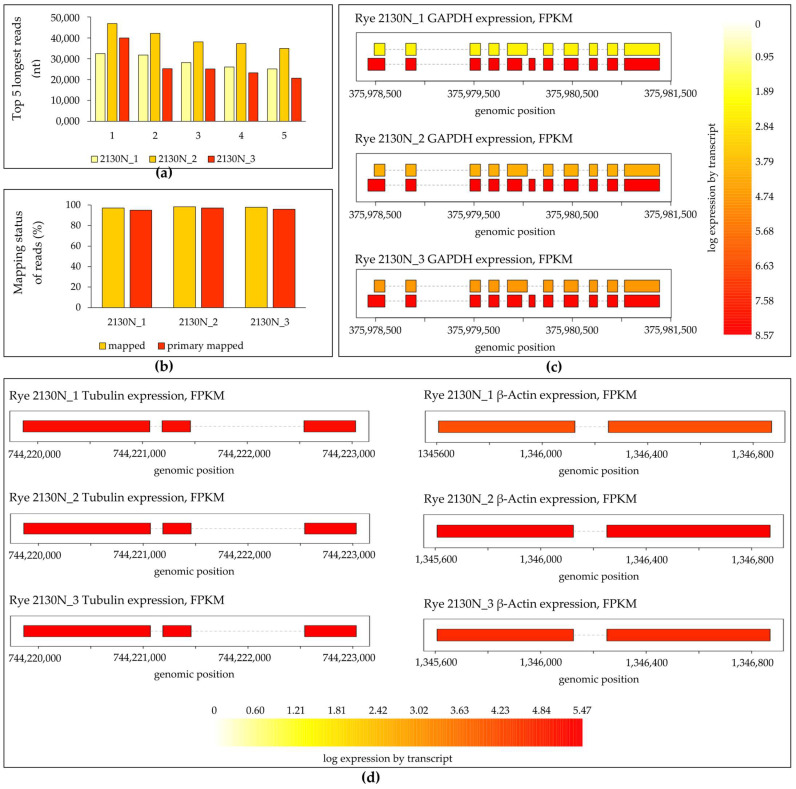
Results of direct cDNA sequencing using nanopore. (**a**) Summary plots for direct cDNA-seq runs with three cumulated biological samples show lists of the top 5 longest reads for each biological replicate; (**b**) results of strand reads mapping to the reference genome of Rye_Lo7_2018_v1p1p1; (**c**) transcript level of *GAPDH* (SECCE6Rv1G0396760), (**d**) *Tubulin* (SECCE5Rv1G0357160), and *β-Actin* (SECCE1Rv1G0000360) in successive biological samples of 2130N rye grains determined by FPKM show log expression by transcript and genomic position.

Three biological replicates of total RNA extracted using protocol B1 were used for mRNA extraction with the Dynabeads mRNA Purification Kit (Thermo Fisher Scientific Baltics UAB, Vilnius, Lithuania), and 100 ng of poli(A) mRNA was used for library preparation with the Direct cDNA sequencing Kit SQK-DCS109 (Oxford Nanopore Technologies, Oxford, UK). Sequencing was performed using the MinION device (Oxford Nanopore Technologies, Oxford, UK) and an R9.4.1 flow cell (Oxford Nanopore Technologies, Oxford, UK). Basecalling was performed with MinKNOW UI 24.02.6 in fast mode. Adapter-trimmed reads were mapped to the rye reference genome Rye_Lo7_2018_v1p1p1 [21] using Minimap2 [22]. The average median read length for all libraries was 812 nucleotides (Table 2), while the longest reads ranged from 20,709 to 46,892 nucleotides (Figure 4a), which agrees with the expected read lengths achieved using ONTs (>30 kb). On average, 97.84% of reads were mapped, and 96.00% were primarily mapped to the reference genome (Figure 4b). Afterwards, we used our mapping results to build a grain transcriptome using StringTie2 [23,24,25]. This allowed the identification of 28.5% novel exons, 23.8% novel introns, and 39.9% novel loci (Table 3).

Analysis of the three selected reference genes, i.e., *Glyceraldehyde-3-phosphate dehydrogenase* (*GAPDH*, SECCE6Rv1G0396760), *Tubulin* (SECCE5Rv1G0357160), and *β-Actin* (SECCE1Rv1G0000360), expressed in rye grains shows the high expression level of the primary *GAPDH* transcript, alongside the lower expression of a novel transcript not present in the reference annotation (Figure 4c). Furthermore, the analysis of *Tubulin* and *β-Actin* in successive biological replicates of the 2130N rye genotype confirmed the transcript structure and expression level, hinting at their potential suitability for use as reference genes in rye grains (Figure 4d).

## 3. Materials and Methods

### 3.1. Plant Material

The experiments were carried out using grains from an inbred line of winter rye (*Secale cereale* L.) 2130N, kindly provided by Irena Kolasińska from IHAR-PIB, Poland. Grains were imbibed in Petri dishes with three layers of filter paper soaked with spring water (Żywiec-Zdrój S.A., Węgierska Górka, Poland) for 48 h at 4 °C, and then germinated in darkness for 72 h at 23 °C. Then, eight seedlings were planted in 12 L pots filled with soil substrate (Hollas, Pasłęk, Poland) and sand (2:1). Plants were cultivated in a phytotron chamber with a 16 h photoperiod at 18 °C during the day and 16 °C at night, with a daylight intensity of 200 µmol photons m^2^ s^−1^ and a humidity of ~70%. Prior to pollination, the ears were isolated using cellophane pollination bags to prevent cross-pollination. Pollen timing was determined on subsequent days, with the day the anthers were shed from the flowers designated as day 0 of pollination. At 21 days after pollination (DAP), grains from the central part of the ear were harvested along with the spikelet hulls (palea and lemma) to optimize total RNA extraction. The collected grains were then frozen in liquid nitrogen and stored at −80 °C until total RNA extraction.

### 3.2. Total RNA Extraction Using Tri Reagent Solution

Total RNA was extracted from rye grains collected 21 days after pollination and stored at −80 °C. For all samples, tissues were ground into a fine powder in liquid nitrogen using sterilized mortars and pestles. In total, 50–75 mg of the powder was homogenized with 1 mL of Tri Reagent Solution (Invitrogen, Waltham, MA, USA) for 5 min at room temperature. The samples were centrifuged at 10,000× *g* for 5 min at 4 °C, and 1 mL of the homogenate was transferred into a new tube.

In the first two total RNA extraction methods (A1 and A2), 100 µL of BCP (Sigma-Aldrich, Schnelldorf, Germany) was added, and the samples were mixed for 10 s using a vortex and then incubated at room temperature for 10 min. Samples were centrifuged at 12,000× *g* for 10 min at 4 °C. The aqueous phase was transferred to a new tube, and either 500 µL of isopropanol (A1) or 250 µL of isopropanol and 250 µL of high-salt precipitation solution (A2) were added. The samples were mixed gently and incubated at room temperature for 5 min and then centrifuged at 8000× *g* for 10 min at 4 °C. The RNA pellet was washed, dried, and dissolved in 100 µL of ultrapure water for molecular biology applications (A&A Biotechnology, Gdańsk, Poland).

In the third method (A3), 1 mL of 96% ethanol was added, the sample was mixed, and then it was centrifuged at 12,000× *g* for 1 min at room temperature. The supernatant was transferred to a new tube, and total RNA was extracted following the Direct-zol RNA Miniprep Kit (Zymo Research Corporation, Irvine, CA, USA) protocol. Total RNA was eluted using 100 µL of ultrapure water for molecular biology applications.

### 3.3. Step-by-Step Protocol for Total RNA Extraction Using LiSTE Buffer and Tri Reagent Solution

1. Freeze rye grains in liquid nitrogen and store −80 °C. Grind into a fine powder in liquid nitrogen using a sterilized mortar and pestle.
2. Suspend 50–75 mg of the powder in 300 µL of LiSTE buffer (150 mM LiCl, 1% SDS, 50 mM Tris-HCl (pH 8.0), and 5 mM EDTA (pH 8.0)). Mix thoroughly and place on ice.
3. Add 300 µL of a mixture of acidic phenol (pH 4.3) (Sigma-Aldrich, Schnelldorf, Germany) and chloroform (1:1). Mix thoroughly and place on ice for 5 min and mix occasionally.
4. Centrifuge at 10,000× *g* for 10 min at 4 °C. Carefully transfer 270 µL of the aqueous phase to a new tube and add 270 µL of chloroform. Mix thoroughly and place on ice for 5 min and mix occasionally.
5. Centrifuge at 10,000× *g* for 10 min at 4 °C. Carefully transfer 200 µL of the aqueous phase to a new tube containing 1 mL of a Tri Reagent Solution. Mix thoroughly and incubate at room temperature for 5 min.
6. Add 100 µL of a BCP, mix for 10 s using a vortex, and incubate at room temperature for 10 min.
7. Centrifuge at 12,000× *g* for 10 min at 4 °C. Carefully transfer 500 µL of the aqueous phase to a new tube and add 500 µL of isopropanol (B1) or 250 µL of isopropanol and 250 µL of high-salt precipitation solution (B2). Mix gently by inverting the tube and incubate at room temperature for 5 min.
8. Centrifuge at 8000× *g* for 10 min at 4 °C. Remove the supernatant and wash the pellet with 1 mL of 75% ethanol.
9. Centrifuge at 7500× *g* for 5 min at 4 °C. Remove the ethanol.
10. Centrifuge at 7500× *g* for 1 min at 4 °C. Remove residual ethanol using a 10 µL pipette. Air-dry the total RNA pellet and dissolve it in 100 µL of ultrapure molecular biology-grade water. Incubate the samples at 4 °C, gently mixing every 15 min until the total RNA pellet is completely dissolved (approximately 1 to 2 h).

### 3.4. Protocol for Total RNA Extraction Using SDS Extraction Buffer, Tri Reagent Solution, and Direct-zol RNA Extraction Kit

Total RNA was extracted as described in Section 3.3 up to stage 5. A total of 1 mL of 96% ethanol was added to 1 mL of homogenate; the sample was mixed thoroughly and centrifuged at 12,000× *g* for 1 min at room temperature (B3). The supernatant was transferred to a new tube, and total RNA was extracted according to the Direct-zol RNA Miniprep Kit protocol, as described in Section 3.2. Total RNA was eluted using 100 µL of ultrapure water.

### 3.5. RT-qPCR Inhibition Test

A total of 1 µg of RNA was treated with DNase I, RNase-free (Invitrogen, Waltham, MA, USA) for 30 min at 37 °C according to the manufacturer’s protocol. The enzyme was then deactivated by adding 50 mM of EDTA and incubating for 10 min at 65 °C. The relative absence of PCR inhibitors in the isolated samples was evaluated by analyzing three isolation replicates. The inhibition test was carried out according to the ENGL guidance document [20]. Each total RNA sample was used for reverse transcription and real-time PCR amplification with ADPRF-specific reference primers, utilizing the Luna Universal One-Step RT-qPCR Kit (New England Biolabs, Ipswich, USA), according to manufacturer’s protocol, on a QuantStudio 5 thermocycler. The protocol included the following steps: reverse transcription step for 10 min at 55 °C, initial denaturation for 1 min at 95 °C, 45 cycles denaturation for 10 s at 95 °C and extension for 30 s at 60 °C, and meld curve calculation between 60 °C and 95 °C. Amplification analysis was calculated from the formula (10^(−1/slope) − 1) × 100.

### 3.6. Library Preparation with Oxford Nanopore Sequencing

Total RNA was extracted from rye grains 14 days after pollination using the B1 protocol. A total of 40 µg of total RNA was treated with DNase I, RNase-free (Thermo Fisher Scientific Baltics UAB, Vilnius, Lithuania) to remove genomic DNA contamination, following the manufacturer’s protocol. mRNA was extracted from the DNase-treated total RNA using the Dynabeads mRNA Purification Kit (Thermo Fisher Scientific Baltics UAB, Vilnius, Lithuania) after the heat inactivation of DNase I. For library preparation, 100 ng of mRNA was used, following the Direct cDNA sequencing—native barcoding (SQK-DCS109 with EXP-NBD114) kit protocol. Sequencing was performed using the MinION device and R9.4.1 flow cell (Oxford Nanopore Technologies, Oxford, UK). Bioinformatic pipeline commands for transcriptome analysis are listed in the Supplementary Materials (Figure S1).

## 4. Conclusions

In conclusion, we report an improvement in the standard Tri Reagent Solution protocol for the isolation of high-quality total RNA from plant tissue rich in polysaccharides. We demonstrate that the extracted mRNA can be successfully utilized for library preparation in Oxford Nanopore Technologies. This optimized protocol for total RNA extraction from the polysaccharide-rich grains of rye has the potential to facilitate transcriptome research in other cereals during their development. The presented modified protocol can be successfully applied to grains of various cereal plant species, wherever RNA purity or quality is a key parameter.

## Figures and Tables

**Table 1:** Total RNA quantity and quality measured by DeNovix DS-11 spectrophotometer after extraction using Tri Reagent Solutions and Direct-zol RNA Miniprep Plus Kit protocols with modifications. Modifications: A1—precipitation with isopropanol; A2—precipitation with isopropanol and high-salt precipitation (IHSP) solution; A3—Direct-zol RNA Miniprep Plus Kit protocol; B1—initial purification before homogenization, followed by precipitation with isopropanol; B2—initial purification before homogenization, followed by precipitation with IHSP solution; B3—initial purification before applying the Direct-zol RNA Miniprep Plus Kit protocol.

Protocols	Concentration (ng/µL)	Absorbance Ratio		Final Concentration Total RNA (µg) per 50 mg
		A_260_/A_280_	A_260_/A_230_	
A1	148–190	1.93–2.04	0.32–0.58	15–19
A2	35–88	1.52–1.83	0.08–0.19	3–9
A3	75–177	1.73–1.84	1.21–1.52	7–18
B1	385–457	1.95–1.96	1.94–2.18	38–46
B2	186–215	2.03–2.09	1.29–1.82	18–21
B3	134–191	1.90–1.92	2.03–2.11	13–19

**Table 2:** Results of direct cDNA-seq for three biological replicates from 2130N grains.

Parameters	2130N_1	2130N_2	2130N_3
Median read length	781.0	894.0	760.0
Median read quality	10.5	10.6	10.5
Number of reads	202,946.0	1,000,582.0	535,489.0
Read length N50	1101.0	1249.0	1067.0
STDEV read length	681.9	724.8	650.6
Total bases	185,553,375.0	1,026,826,650.0	486,986,614.0
Total bases aligned	127,181,617.0	733,471,879.0	336,523,718.0

**Table 3:** Number of novel exons, introns, and loci identified in direct cDNA-seq in comparison to Rye_Lo7_2018_v1p1p1 [20].

Feature	Novel/All	Novel Feature Percentage
Exons	62,253/218,514	(28.5%)
Introns	37,094/155,685	(23.8%)
Loci	22,823/57,167	(39.9%)

## Data Availability

The data presented in this study will be openly available in NCBI SRA at https://www.ncbi.nlm.nih.gov/sra/PRJNA1195027 (6 December 2024); reference data, PRJNA1195027; temporary submission ID: SUB14913406; release date: 6 December 2024.

## Supplementary Materials

The following supporting information can be downloaded at: https://www.mdpi.com/article/10.3390/ijms252313188/s1.
