# Supplementary material for: An Improved RNA Extraction Protocol for Rye Grain Full-Length Transcriptome Sequencing
Source: Int J Mol Sci. 2024 Dec 8;25(23):13188. doi: 10.3390/ijms252313188 (PMC11642000; doi:10.3390/ijms252313188)
Supplement: Supplementary file 1 [file ijms-25-13188-s001.zip › Figure S1.pdf]

Bioinformatic pipeline commands:

1) File filtering on called fastq files

```
pyBioTools Fastq Filter \
-i $f1 \
-o filtered_$f1 \
--remove_duplicates \
--min_len 100 \
--min_qual 7
```

2) Prepare a minimap2 index and map the reads, obtain mapping statistics and convert to sorted bams

```
minimap2 -d lo7.mmi GCA_902687465.1_Rye_Lo7_2018_v1p1p1_genomic_gtf_corrected.fa
```

```
minimap2 -a -x splice -k14 -uf -t 64 \
lo7.mmi \
$f1 > $f2.sam
```

```
samtools flagstat $f1
```

```
samtools sort -@ 32 $f1 |
samtools view -@ 32 -b -o $f2".bam"
```

3) Detect novel transcript variants using stringtie2

```
stringtie -L -G ../Secale_cereale.Rye_Lo7_2018_v1p1p1.59.gff3 \
-o $f2 $f1
```

```
stringtie --merge \
-G Secale_cereale.Rye_Lo7_2018_v1p1p1.59.gff3 \
-l SCEREALE192 \
-o 2130N.gtf \
2130N_1.gtf 2130N_2.gtf 2130N_3.gtf
```

```
gffcompare -r Secale_cereale.Rye_Lo7_2018_v1p1p1.59.gff3 \
-V \
-R \
-s GCA_902687465.1_Rye_Lo7_2018_v1p1p1_genomic_gtf_corrected.fa \
2130N.gtf
```

4) Prepare Ballgown count tables

```
stringtie -eB \
-o $1 \
-G gffcmp.annotated.gtf \
$f1.bam
```
