# Supplementary material for: An Improved RNA Extraction Protocol for Rye Grain Full-Length Transcriptome Sequencing
Source: Int J Mol Sci. 2024 Dec 8;25(23):13188. doi: 10.3390/ijms252313188 (PMC11642000; doi:10.3390/ijms252313188)
Supplement: Supplementary file 1 [file ijms-25-13188-s001.zip › Table S1.pdf]

Table S1. Total RNA quantity and quality measured by Agilent 2100 Bioanalyzer spectrophotometer after extraction using Tri Reagent Solutions and Direct-zol RNA Miniprep Plus Kit protocols with modifications. Modifications: A1 – precipitation with isopropanol; A2 – precipitation with isopropanol and high-salt precipitation (IHSP) solution; A3 – Direct-zol RNA Miniprep Plus Kit protocol; B1 – initial purification before homogenization, followed by precipitation with isopropanol; B2 – initial puri-fication before homogenization, followed by precipitation with IHSP solution; B3 – initial purifica-tion before applying the Direct-zol RNA Miniprep Plus Kit protocol.

Protocols	Replication	RNA Area	RNA Concentration [ng/μL]	rRNA Ratio [28S / 18S]	RNA Integrity Number	18S				28S			
						Start Size [nt]	End Size [nt]	Area	% of Total Area	Start Size [nt]	End Size [nt]	Area	% of Total Area
A1	1	304.2	140	1.6	N/A	1.621	2.209	15.1	5	2.591	3.474	24.3	8
	2	358.6	165	1.8	N/A	1.595	2.166	24.3	6.8	2.478	3.319	43.8	12.2
	3	218.9	150	1.4	N/A	1.577	2.102	11.2	5.1	2.576	3.551	15.3	7
A2	1	22.2	10	0	2.3	n/a	n/a	n/a	n/a	n/a	n/a	n/a	n/a
	2	25.3	12	0.7	7.9	1.632	2.141	2.8	10.9	2.672	3.191	1.9	7.6
	3	27.1	19	0	2.6	n/a	n/a	n/a	n/a	n/a	n/a	n/a	n/a
A3	1	93.4	43	1.7	N/A	1.578	1.966	8	8.6	2.454	3.333	13.3	14.2
	2	258.4	119	1.8	9.7	1.556	1.94	45.2	17.5	2.188	3.268	83.2	32.2
	3	91.8	63	1.5	N/A	1.531	1.916	9.7	10.6	2.326	3.287	14.7	16
B1	1	696.9	320	1.7	9.5	1.617	2.057	131	18.8	2.298	3.43	225.7	32.4
	2	699.9	321	1.8	9.8	1.568	2.028	146.2	20.9	2.211	3.359	268.9	38.4
	3	460.5	315	1.7	9.5	1.579	2.043	79.8	17.3	2.289	3.385	133.1	28.9
B2	1	337	155	1.9	9.7	1.591	2.167	80.9	24	2.346	3.528	150.4	44.6
	2	443.7	204	1.9	9.6	1.547	2.124	101.4	22.9	2.444	3.427	189.1	42.6
	3	235.3	161	1.8	9.5	1.551	2.094	51.4	21.8	2.314	3.452	90	38.3
B3	1	246.1	113	1.9	N/A	1.605	2.143	39.2	15.9	2.442	3.387	75.9	30.8
	2	388	178	1.7	N/A	1.557	2.088	64.9	16.7	2.112	3.29	113.1	29.1
	3	215.6	148	1.8	N/A	1.559	2.0557	30	13.9	2.277	3.28	53.4	24.8
